# Supplementary material for: Functional Dissection of P1 Bacteriophage Holin-like Proteins Reveals the Biological Sense of P1 Lytic System Complexity
Source: Int J Mol Sci. 2022 Apr 11;23(8):4231. doi: 10.3390/ijms23084231 (PMC9025707; doi:10.3390/ijms23084231)
Supplement: Supplementary file 1 [file ijms-23-04231-s001.zip › Figure_S1.pdf]

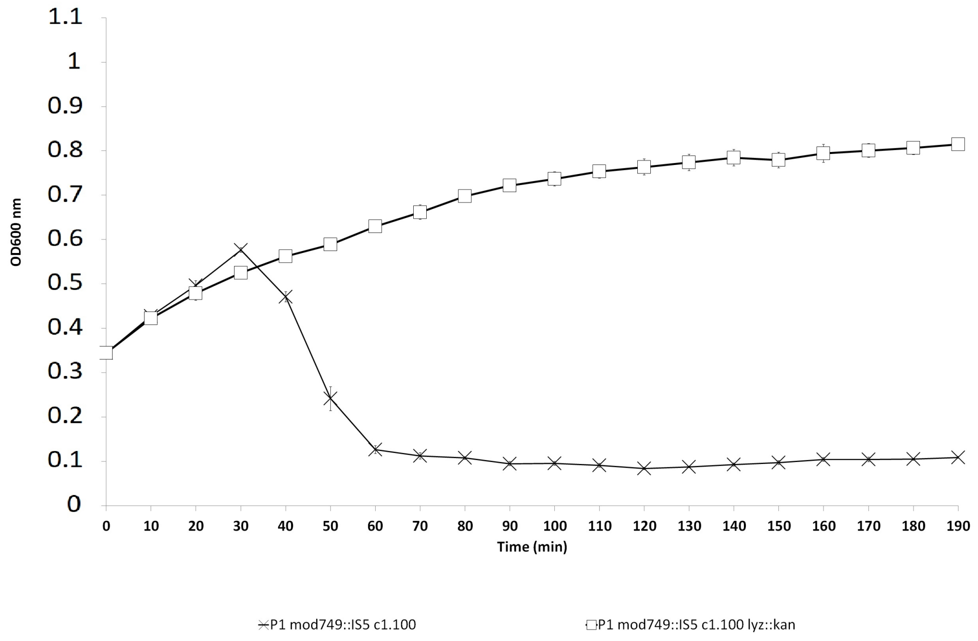

Figure S1. Changes in the optical density of cultures of *E. coli* cells containing wild-type P1 or the P1 mutant depleted of the endolysin gene (*lyz*), upon induction of P1 lytic development. The graph shows the average results of measurement of changes in the optical density of cell cultures in at least three independent experiments. The legend applies to both charts. The bars represent the standard deviation.
