# Supplementary material for: Functional Dissection of P1 Bacteriophage Holin-like Proteins Reveals the Biological Sense of P1 Lytic System Complexity
Source: Int J Mol Sci. 2022 Apr 11;23(8):4231. doi: 10.3390/ijms23084231 (PMC9025707; doi:10.3390/ijms23084231)
Supplement: Supplementary file 1 [file ijms-23-04231-s001.zip › Table_S1_plasmids.pdf]

Table S1. Plasmids used in this study

Plasmid[a]	Cloned genes	Description (comments)[b]	Replication origin /Selective marker	Source or reference
pGBT30	*tacPO korB*	Expression plasmid containing 1.1 kb DNA insert in place of EcoRI-SalI fragment of *lac* operon promoter	pMB1/amp^R	1
pGB2	-	Mini-pSC101-derived plasmid vector	pSC101/spec^R	2
pACYC184	-	Cloning vector	p15A/cm^R	3
pRPG18	*trpR*	A derivative of pACYC184 cloning vector containing the *E. coli trpR* gene	p15A/cm^R	3
pMLO211	*trpR*	A derivative of pSC101 cloning vector containing the *E. coli trpR* gene; constructed by the insertion of BamHI-BamHI fragment of pRPG18 plasmid encoding *trpR* into BamHI-digested pGB2	pSC101/spec^R	This study
pUC4K	kan^R	A multicopy *E. coli* vector containing the kanamycin resistance cassette of Tn*903*	pMB1/amp^R, kan^R	4
pUCP7/1223	*'lyz, lydD, lydE*	A derivative of pUC18 plasmid vector with a cloned fragment of P7 genomic library containing the 3' fragment of *lyz* gene and the whole *lydD* and *lydE* genes	pMB1/amp^R	Plasmid collection of IBB PAS
pUCP7/679	*lyz*	A derivative of pUC18 plasmid vector with a cloned fragment of P7 genomic library containing the *lyz* gene	pMB1/amp^R	Plasmid collection of IBB PAS
pUCP1/546	*cin, lydC, lydA*	A derivative of pUC18 plasmid vector with a cloned fragment of P1 genomic library containing the *cin* and *lydC* genes and the 5' fragment of *lydA*	pMB1/amp^R	5
pUCP1/257	*lydC, lydA, hdf, darA'*	A derivative of pUC18 plasmid vector with a cloned fragment of P1 genomic library containing the *lydA*, *lydB* and *hdf* genes and the 5' fragment of *darA*	pMB1/amp^R	5
pBEF111	*trpPO parA*	4.1 kb, pBR322-derivative containing the P1 *parA* gene cloned under the control of *Serratia marcescens* promoter-operator region of tryptophan operon (*trpPO*)	pMB1/amp^R	6
pBEF119	*trpPO parAB*	A derivative of pBR327 cloning vector carrying P1 *parAB* genes under the control of *trpPO*	pMB1/amp^R	6
pMLO24	*trpPO parA*	A derivative of pBEF111 plasmid with the SalI recognition site at the end of *parA* gene replaced by the HindIII recognition site; constructed by the insertion of HindIII linker (CCAAGCTTGG) in the SalI-digested pBEF111, with sticky ends filled in with the Klenow fragment of DNA polymerase I	pMB1/amp^R	This study
pMLO29	*trpPO parAB*	A derivative of pBEF119 containing the *parAB* genes of P1 cloned under the control of *trpPO* promoter; constructed by the insertion of BamHI linker (CGGGATCCCG) in the EcoRI-digested pBEF119 with sticky ends filled in with the Klenow fragment of DNA polymerase I	pMB1/amp^R	This study
pMLO30	*trpPO parAB*	A derivative of pACYC184 containing the *parAB* genes of P1 cloned under the control of *trpPO*	p15A/cm^R	This study
pACE1	*trpPO parA'*, *'lyz, lydD, lydE*	A derivative of pMLO24 containing the 5' end of *parA* gene, the 3' end of *lyz* gene and the *lydD* and *lydE* genes of P7. Constructed by the insertion of SphI-DraIII fragment of pUCP7/1223 (blunted at the DraIII end) in SphI-SalI-cleaved pMLO24 (blunted at the SalI end).	pMB1/amp^R	This study
pACE2	*trpPO parA', lyz, lydD, lydE*	A derivative of pACE1 containing P1 *lyz*, *lydD* and *lydE* genes. Constructed by the insertion of SmaI-SphI fragment of pUCP1/679 containing the P1 *lyz* gene trunkated at the 3' end into the SphI-XhoI cleaved pACE1 (blunted at the XhoI end).	pMB1/amp^R	This study
pACE3	*trpPO lyz, lydD,*	A derivative of pACE2 containing *lyz*, *lydD* and *lydE* genes of P1	pMB1/amp^R	This study

	lydE	under the control of *trp* promoter-operator region, and depleted of P1 *parA* gene. Constructed by the religation of large HindIII-KpnI fragment of pACE2 (blunted at both ends).		
pACE4	*trpPO lyz*	A derivative of pACE3 depleted of 161 nucleotide residues from the 3' end of *lydD* gene and the *lydE* gene. Constructed by the digestion of pACE3 with Esp3I and Eco47III and religation of the repaired ends of the larger fragment.	pMB1/amp^R	This study
pACE5	*trpPO lyz, lydD*	A derivative of pACE3 depleted of the 3' part of *lydE* gene. Constructed by the digestion of pACE3 with HpaI and Eco47III and religation of the repaired ends of the larger fragment.	pMB1/amp^R	This study
pACE10	*trpPO* R_{λ}	A derivative of pMLO30 containing the phage λ *R* gene in place of phage P1 *parAB* genes. Constructed by the insertion of phage λ genome fragment (pos. 45458-46021) amplified with primers oMLO322 and oMLO323 and digested with HindIII and SalI in place of HindIII-SalI fragment containing *parAB* genes.	p15A/cm^R	This study
pWWO1	*tacPO lyz*	A derivative of pACE10 with HindIII-SalI fragment containing the phage λ *R* gene replaced with P7 *lyz* gene. Constructed by the insertion of P7 genome fragment (pos. 24082-24635) amplified with the oMLO391 and oMLO392 primers and digested with HindIII and SalI, in place of HindIII-SalI fragment of pACE10 obtained after digestion with SalI and partial digestion with HindIII.	p15A/cm^R	This study
pWWO2	*trpPO lyz::kan^R, lydD, lydE*	A derivative of pACE3 containing the kan^R cassette in the *lyz* gene. Constructed by the insertion of kan^R cassette of pUC4K amplified with oMLO607 and oMLO608 primers and digested with PaeI, in PaeI site of pACE3 *lyz* gene.	pMB1/amp^R, kan^R	This study
pAKI1	*trpPO lyz, lydD::kan^R, lydE*	A derivative of pACE3 containing the kanamycin resistance (kan^R) cassette in the *lydD* gene. Constructed by the insertion of kan^R cassette of pUC4K amplified with oAKI1 and oAKI2 primers and digested with Esp3I, in Esp3I site of pACE3 *lydD* gene.	pMB1/amp^R, kan^R	This study
pAKI2	*cin, lydCΔ12_13TG, lydA'*	A derivative of pUCP1/546 with the *lydC* gene inactivated by the deletion of two nucleotides at pos. 12 and 13 of *lydC* gene. Constructed by digestion of pUCP1/546 with RseI, blunting extended 3' ends and religation.	pMB1/amp^R	This study
pAKI3	*lydC, lydA::195_196GATC, hdf, darA'*	A derivative of pUCP1/257 with the *lydA* gene inactivated by the insertion of four nucleotides at pos. 195-196 of *lydA*. Constructed by digestion of pUCP1/257 with BglII, filling in extending 5' termini and religation.	pMB1/amp^R	This study
pAKI6	*lydD*	A derivartive of pGBT30 in which the EcoRI-SalI fragment containing the RK2 plasmid *korB* gene was replaced with the P1 genome fragment from pos. 20074-20309 containing the *lydD* gene with the SD sequence of *lydA* gene. Constructed by the insertion of the P1 genome fragment amplifield with the oAKI18 and oAKI19 primers and digested with EcoRI and SalI, in place of EcoRI-SalI fragment of pGBT30	pMB1/amp^R	This study
pAKI8	*ssb*	A derivartive of pGBT30 with the EcoRI-SalI fragment containing the RK2 plasmid *korB* gene replaced with the P1 genome fragment from pos. 20903-21549 containing the regulatory region of *lyz* and *ssb* genes and the *ssb* gene. Constructed by the insertion of the P1 genome fragment amplifield with the oAKI22 and oAKI24 primers and digested with EcoRI and SalI, in place of EcoRI-SalI fragment of pGBT30.	p15A/cm^R	This study
pAKI9	*lydD*	A derivative of pAKI8 in which the MfeI-SnaBI fragment was replaced with the P1 genome fragment (pos. 20074-20355) containing the *lydD* gene. Constructed by the insertion of the P1 genome fragment amplifield with the oAKI20 and oAKI37 primers and digested with MfeI and SnaBI, in place MfeI-SnabI fragment of pAKI8.	p15A/cm^R	This study
pAKI13	R_{λ}, *lydD*	A derivative of pAKI9 in which the SalI-MfeI fragment was replaced with the phage λ genome fragment (pos. 45493-45969) containing the *R* gene and preceded by the 20 nucleotide region	pMB1/amp^R	This study

		containing the SD sequence of P1 *lyz* gene. Constructed by the insertion of the phage λ genome fragment amplifield with oAKI35 an oAKI38 primers and digested with SalI and MfeI, in place SalI-MfeI fragment of pAKI9.		
pAKI21	*'pro, lyz*	A derivative of pGBT30 in which the EcoRI-SalI fragment containing the RK2 plasmid *korB* gene was replaced with the two connected P1 genome fragments (pos. 19566-20040 and 20318-20693) containing the fragment of *pro* gene and the *lyz* gene, respectively. Constructed by the replacement of EcoRI-SalI fragment of pGBT30 containing the RK2 plasmid *korB* gene with the two ligated P1 genome fragments amplifield with the oAKI40 and oAKI50, and oAKI42 and oAKI43 primers and digested with EcoRI and SacI and SacI and SalI, respectively.	pMB1/amp^R	This study
pAKI23	R_λ, *lydE*	A derivative of pAKI13 in which the MfeI-SnaBI fragment was replaced with the P1 genome fragment (pos. 19800-20251) containing the *lydE* gene and its regulatory region and a fragment of *pro* gene. Constructed by the insertion of the P1 genome fragment amplifield with the oAKI55 and oAKI56 primers and digested with SnaBI and MfeI, in place SnaB-MfeI fragment of pAKI13.	pMB1/amp^R	This study
pAKI26	*lyz, lydDΔ16-81, lydE*	A derivative of pAKI21 in which the SacI-EcoRI fragment containing the fragment of P1 *pro* gene was replaced with the P1 genome fragment (pos. 19800-20251) containing the *lydE gene.* Constructed by the replacement of SacI-EcoRI fragment of pAKI21 with the P1 genome fragments amplifield with the oAKI59 and oAKI60 primers and digested with SacI and EcoRI.	pMB1/amp^R	This study

[a] The sequence of P7 Lyz protein differs by one, non-essential amino acid residue (V to L replacement at pos. 183 of 185) from that of P1 Lyz, and the sequences of P1 and P7 LydD proteins are identical, so that we used *lyz* and *lydD* genes amplified from the P1 and P7 DNA replaceably.
[b] Positions of sequences derived from the P1 and λ bacteriophage genomes refer to sequences deposited in GenBank under the accession number AF234172.1 and NC_001416.1, respectively.
SD: Shine-Dalgarno sequence

References:

1. Jagura-Burdzy, G.; Macartney, D.P.; Zatyka, M.; Cunliffe, L.; Cooke, D.; Huggins, C.; Westblade, L.; Khanim, F.; Thomas, C.M. Repression at a Distance by the Global Regulator KorB of Promiscuous IncP Plasmids. *Mol. Microbiol.* **1999**, *32*, 519–532. https://doi.org/10.1046/j.1365-2958.1999.01365.x.

2. Churchward, G.; Belin, D.; Nagamine, Y. A PSC101-Derived Plasmid Which Shows No Sequence Homology to Other Commonly Used Cloning Vectors. *Gene* **1984**, *31*, 165–171. https://doi.org/10.1016/0378-1119(84)90207-5.

3. Sambrook, J.; Fritsch, E.F.; Maniatis, T. *Molecular Cloning: A Laboratory Manual*, 2nd. ed.; Cold Spring Harbor Laboratory Press: Cold Spring Harbor, NY, USA, 1989

4. Taylor, L.A.; Rose, R.E. A Correction in the Nucleotide Sequence of the Tn903 Kanamycin Resistance Determinant in PUC4K. *Nucl. Acids Res.* **1988**, *16*, 358–358. https://doi.org/10.1093/nar/16.1.358.

5. Łobocka, M.B.; Rose, D.J.; Plunkett, G.; Rusin, M.; Samojedny, A.; Lehnherr, H.; Yarmolinsky, M.B.; Blattner, F.R. Genome of Bacteriophage P1. *J. Bacteriol.* **2004**, *186*, 7032–7068. https://doi.org/10.1128/JB.186.21.7032-7068.2004.

6. Funnell, B.E. Mini-P1 Plasmid Partitioning: Excess ParB Protein Destabilizes Plasmids Containing the Centromere ParS. *J. Bacteriol.* **1988**, *170*, 954–960. https://doi.org/10.1128/jb.170.2.954-960.1988.
