# Supplementary material for: Functional Dissection of P1 Bacteriophage Holin-like Proteins Reveals the Biological Sense of P1 Lytic System Complexity
Source: Int J Mol Sci. 2022 Apr 11;23(8):4231. doi: 10.3390/ijms23084231 (PMC9025707; doi:10.3390/ijms23084231)
Supplement: Supplementary file 1 [file ijms-23-04231-s001.zip › Table_S2_oligonucleotides.pdf]

Tabela S2. Oligonucleotides used in this work

**Name**	**Sequence[a] 5'-3'**	**Tm [°C]**	**Complementary region[b]**	**Aim of use**
oMLO322	atataataaagcttCAAACGCTTCGCTGCTAAA	56.6/p	Phage λ genome, pos. 45458-45476	Amplification of bacteriophage λ *R* gene and addition of HindIII and SalI recognition sites at its flanks
oMLO323	ataataagtcgacAGGCAGACGATGATGCAGATA	56.6/p	Phage λ genome, pos. 46001-46021	
oMLO391	atataagcttGTAAAAAATTCGCTTATGAGGTCTTTATGAA	58/p	Phage P7 *c1-100 mod749*::IS*5* IS*1*::Tn*9* genome, pos. 24635-24665	Amplification of *lyz* gene of phage P7 and addition of HindIII and SalI recognition sites at its flanks
oMLO392	ttaagtcgacTTATTCATTGATCAGCCCCGTCAG	58/p	Phage P7 *c1-100 mod749*::IS*5* IS*1*::Tn*9* genome, pos. 24082-24105	
oMLO607	attctgcatgcGCGCTGAGGTCTGCCTCGTGAAGA	51.6/p	pUC4K plasmid, pos. 433-456	Amplification of Kan^{R} cassette of pUC4K plasmid and addition of PaeI recognition sites at its flanks
oMLO608	attcagcatgcAAAGCCACGTTGTGTCTCAAAATC	51.6/p	pUC4K plasmid, pos. 1622-1650	
oMLO597	CACGCTGATCGCTAAAACATTGTGC	59,3/p	pWWO1 plasmid, pos.	Verification of sequence correctnes of pWWO1 plasmid insert (P7 *lyz* gene)
oMLO598	GGAAGGAGCTGACTGGGTTGAAGG	59,3/p	pWWO1 plasmid, pos.	
oAKI1	atattcaatgagacgGCGCTGAGGTCTGCCTCGTGAAGA	68,7	pUC4K plasmid, pos. 433-456	Amplification of Kan^{R} cassette of pUC4K plasmid
oAKI2	atatcgtctcaTTGAAAAGCCACGTTGTGTCTCAAAATC	64,0	pUC4K plasmid, pos. 1622-1650	
oAKI4	GTCACTGCAACAAAGATCTCCGTA	61,1	Phage P1 *c1-100 mod749*::IS*5* IS*1*::Tn*9* genome, pos. 31035-31058	Identification of *lydCΔ12_13TG* mutation in the genome of P1 *c1-100 mod749*::IS*5* IS*1*::Tn*9* recombinants
oAKI5	CCAGCACAACTAAGTGACGCAT	61,4	Phage P1 *c1-100 mod749*::IS*5* IS*1*::Tn*9* genome, pos. 31820-31843	
oAKI6	ACCCATACACAGGAAATTCTTAACACAGA	62,6	Phage P1 *c1-100 mod749*::IS*5* IS*1*::Tn*9* genome, pos. 19320-19348	Confirmation of Kan^{R} insertion in the *lydD* gene of P1 *c1-100 mod749*::IS*5* IS*1*::Tn*9* recombinants
oAKI7	AGACCTGAGAACCTTTTCGTAAATACTCACT	63,9	Phage P1 *c1- 100 mod749*::IS*5* IS*1*::Tn*9* genome, pos. 21255-21285	
oAKI8	CTTTCGTTGTTGCGCGGTTGCTGGAGTC	70,5	Phage P1 *c1-100 mod749*::IS*5* IS*1*::Tn*9* genome, pos. 30292-30319	Identification of *lydA::195GATC* mutations in the genomes of P1 *c1-100 mod749*::IS*5* IS*1*::Tn*9* recombinants
oAKI9	GCATGCTGCGCCCAAGACGGTCTAACTT	71	Phage P1 *c1- 100 mod749*::IS*5* IS*1*::Tn*9* genome, pos. 31843-31870	
oAKI10	CAAGTTCTAAATGCGTGATGTCCT	59,6	Phage P1 *c1-100 mod749*::IS*5* IS*1*::Tn*9* genome, pos. 30770-30793	Confirmation of the *lydCΔ12TG* mutation presence in a phage recombinat by sequencing
oAKI11	CGATAATAAATACCGCCGTTCTTA	57,1	Phage P1 *c1-100 mod749*::IS*5* IS*1*::Tn*9* genome, pos. 33452-33475	
oAKI12	CCGATCGCATCTGCCATGTCCACACCCT	71,9	Phage P1 *c1-100 mod749*::IS*5* IS*1*::Tn*9* genome, pos. 28730-28757	Confirmation of the *lydA::195GATC* mutation presence in a phage recombinant by sequencing. Used with OAKI9.

oAKI18	ttaagtcgacACTATTATCGCCTTCGCCTGCGTG	65,6	Phage P1 *c1-100 mod749*::IS*5* IS*1*::Tn*9* genome, pos. 20074-20097	Amplification of phage P1 *c1-100 mod749*::IS*5* IS*1*::Tn*9* genome fragment of coord. 20074-20332
oAKI19	tatagaattc**GTGAAAAAGGGAGGCGGGAA**ATGAATAAACTCCGGCAGCTCCGC	72,2	Phage P1 *c1-100 mod749*::IS*5* IS*1*::Tn*9* genome, pos. 20309-20332	
oAKI22	tatagtcgacTTTACGCTCCAGGACAAACAC	65	Phage P1 *c1-100 mod749*::IS*5* IS*1*::Tn*9* genome, pos. 20903-20923	Amplification of phage P1 *c1-100 mod749*::IS*5* IS*1*::Tn*9* genome fragment of coord. 21549-20903
oAKI24	tatagaattcCCGCTTTTCTTTCAGTCGTTT	62,3	Phage P1 *c1-100 mod749*::IS*5* IS*1*::Tn*9* genome, pos. 21549-21569	
oAKI35	tatagtcgac**aattcgcttatgaggtcttt**ATGGTAGAAATCAATAATCAAC	65,2	Phage λ genome, pos. 45493-45514	Amplification of phage λ *R* gene and supplementing it with the SD sequence of P1 *lyz*
oAKI38	tatacaattgTCATACATCAATCTCTCTGA	57,2	Phage λ genome, pos. 45950-45969	
oAKI20	tatatacgtaACTATTATCGCCTTCGCCTGCGTG	44,12	Phage P1 *c1-100 mod749*::IS*5* IS*1*::Tn*9* genome, pos. 20074-20097	Amplification of phage P1 *lydD* gene with its own SD sequence
oAKI37	tatacaattgGCTTTGCCTGACGGGACTGGTC	50	Phage P1 *c1-100 mod749*::IS*5* IS*1*::Tn*9* genome, pos. 200334-20355	
oAKI55	tataTACGTAGAACTGGATAACCTCCCGATTTTC	38,24	Phage P1 *c1-100 mod749*::IS*5* IS*1*::Tn*9* genome, pos. 19800-19823	Amplification of phage P1 *c1-100 mod749*::IS*5* IS*1*::Tn*9* genome fragment of coord. 19800-20251
oAKI56	tataCAATTGGCGGTATATGTGCTCAATGAGACG	41,18	Phage P1 *c1-100 mod749*::IS*5* IS*1*::Tn*9* genome, pos. 20228-20251	
oAKI40	tatagaattcCATGGGTACTCCGACGAGTC	64,9	Phage P1 *c1-100 mod749*::IS*5* IS*1*::Tn*9* genome, pos. 19556-19576	Amplification of phage P1 *c1-100 mod749*::IS*5* IS*1*::Tn*9* genome fragment of coord. 19556-20040
oAKI50	tatagagctcTGGTGGTTATTTAAACGGATTGATTGA	63,5	Phage P1 *c1-100 mod749*::IS*5* IS*1*::Tn*9* genome, pos.20013- 20040	
oAKI42	tatagagctcCCGGAGTTTATTCATTGACC	63,5	Phage P1 *c1-100 mod749*::IS*5* IS*1*::Tn*9* genome, pos. 20318-20338	Amplification of phage P1 *c1-100 mod749*::IS*5* IS*1*::Tn*9* genome fragment of coord. 20318-20693
oAKI43	tatagtcgacTGACGGGATCGGTAATACAC	64,5	Phage P1 *c1-100 mod749*::IS*5* IS*1*::Tn*9* genome, pos. 20673-20693	
oAKI59	tatagagctcGCGGTATATGTGCTCAATGAGACG	66,3	Phage P1 *c1-100 mod749*::IS*5* IS*1*::Tn*9* genome, pos. 20228-20251	Amplification of phage P1 *c1-100 mod749*::IS*5* IS*1*::Tn*9* genome fragment of coord. 19800-20251
oAKI60	tatagaattcGAACTGGATAACCTCCCGATTTTC	63,5	Phage P1 *c1-100 mod749*::IS*5* IS*1*::Tn*9* genome, pos. 19800-19824	

[a]Fragments of primers complementary to the target sequences are in capital letters, the fragment of oAKI35 primer with the SD sequence of *lyz* gene is in bold.

[b]Positions in the genomes of plasmid pUC4K and phages P7 *c1.9*ts, P1 *c1-100 mod749*::IS*5* IS*1*::Tn*9* and λ refer to the sequences deposited in GenBank under the accession numbers X06404.1, AF503408.1, AF234172.1 and NC_001416.1, respectively.
